# Species‐specific environmental DNA analysis of the index species in soil ecosystem, *Allonychiurus kimi* (Collembola: Onychiuridae)

**DOI:** 10.1002/ece3.9598

**Published:** 2022-12-12

**Authors:** Yun‐Sik Lee, Minhyung Jung, June Wee, Yongeun Kim, Doo‐Hyung Lee, Dong‐Sung Lee, Taewoo Kim, Kijong Cho, Cheolho Sim

**Affiliations:** ^1^ Department of Biology Baylor University Waco Texas USA; ^2^ Ojeong Eco‐Resilience Institute Korea University Seoul South Korea; ^3^ Department of Life Science Gachon University Seongnam South Korea; ^4^ BK21 FOUR R&E Center for Environmental Science and Ecological Engineering Korea University Seoul South Korea; ^5^ Department of Life Science University of Seoul Seoul South Korea; ^6^ Division of Environmental Science and Ecological Engineering Korea University Seoul South Korea

**Keywords:** eDNA‐based biomonitoring, extraorganismal eDNA, intraorganismal eDNA, soil‐dwelling microinvertebrate

## Abstract

Collembola are abundant and have significant roles in the soil ecosystem. Therefore, the phenotypic endpoints of Collembola population or community have been used as an effective bioindicator for assessing soil quality. Since the identification and counting the collembolans in the soil is a laborious and costly procedure, environmental DNA (eDNA)‐based biomonitoring was proposed as an analysis tool of collembolan species found in the soil. In this study, standard primer sets for the species‐specific eDNA analysis using *Allonychiurus kimi*, a soil bioindicator species was selected. Then, the primers were tested for specificity and sensitivity from the soil samples. Two different eDNA samples were tested: (1) eDNA samples were extracted from the soil with *A. kimi* individuals (intra‐organismal eDNA). (2) The samples from the soil without *A. kimi* individuals (extra‐organismal eDNA). The two primers were confirmed in their sensitivity and specificity to the two types of eDNA samples selected. *C*_t_‐values from both intra‐ and extra‐organismal eDNA showed the significant correlations to the number of inoculated *A. kimi* (adj. *R* ^2^ = 0.7453–0.9489). These results suggest that in excretion, egg, and other exuviae had a significant effect on eDNA analysis from soil samples taken. Furthermore, our results suggest that environmental factors should be considered when analyzing eDNA collected from soil.

## Graphical abstract

In this study, standard primer sets for the species‐specific eDNA analysis using *Allonychiurus kimi*, a soil bioindicator species was selected. Then, the primers were tested for specificity and sensitivity from the soil samples. Two primers were confirmed in their sensitivity and specificity to the two eDNA samples selected. *C*_t_‐values from both intra‐ and extra‐organismal eDNAs showed the significant correlations to the number of inoculated *A. kimi*. These results suggest that in excretion, egg, and other exuviae had a significant effect on eDNA analysis from soil samples taken.

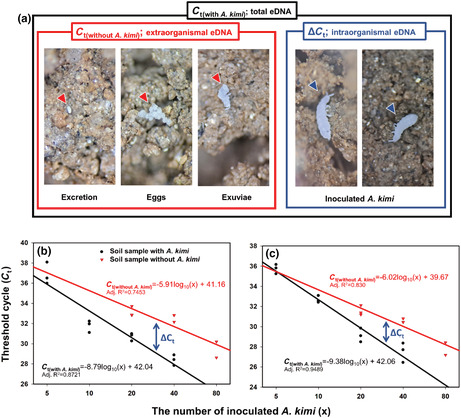

## 1 INTRODUCTION

Collembola are small arthropods that play a significant influence on soil ecosystems by feeding on dead organic matter and soil microorganisms (Hopkin, 1997). Most of the species in Collembola have a limited mobility due to lack of wings which make it difficult for them to migrate to different habitats (Bengtsson et al., 1994). Since it is difficult to escape from an original habitat, Collembola are constantly affected by their soil conditions, which include biological and environmental factors. This makes them a useful index species that can be used to evaluate chemical toxicity and risk analysis for pollutants in soil ecosystem (Fountain & Hopkin, 2005; Lee, Kim, et al., 2021). Therefore, many researchers have used the major collembolan species to evaluate the soil quality from different environmental conditions (Lee, Son, et al., 2019; Wee, Lee, Kim, Lee, et al., 2021).

In in situ and ex situ experiments using Collembola, the phenotypic index, such as the number of adults and juveniles (abundance), head capsule width, and weight of body have been considered as key indices representing their fitness in the inhabited soil (Lee, Son, et al., 2019; Lin et al., 2019; Wee, Lee, Kim, Lee, et al., 2021). However, most Collembola species have a small body size (1–2 mm) and have negative phototaxis behavior which drives the individuals to burrow into soil (Salmon & Ponge, 1998; Schaller, 1972). Therefore, investigating these phenotypic indices mainly relies on manual eye‐counting under a microscope, which is a labor‐intensive and costly procedure (OECD, 2008).

Environmental DNA (eDNA) approach provides new avenues of biomonitoring (Rees et al., 2014). To date, researchers have utilized these approaches to detect target organisms, estimate species biomass from samples, and development indices for assessing the disturbance in aquatic environments (Graham et al., 2019; Knudsen et al., 2019; Kodama et al., 2022; Laramie et al., 2015; Scriver et al., 2015). However, eDNA‐based biomonitoring has not been as widely used for the terrestrial environment in the same way (Katz et al., 2021; Saitoh et al., 2016; Yasashimoto et al., 2021). Pawlowski et al. (2021) have suggested that an optimization of eDNA analytical method or standardization needs to be developed in order to improve the terrestrial biomonitoring for certain taxa.

Among eDNA‐based biomonitoring methods, a species‐specific detection method based on real‐time quantitative polymerase chain reaction (qPCR) can be applied by researchers with little cost and effort and it is also an easy method for eDNA detection standardization (Heid et al., 1996; Laramie et al., 2015; Tsuji et al., 2019). Many researchers employ this technique for eDNA quantification since it enables the detection of short species‐specific eDNA in various contexts (Mauvisseau et al., 2019; Rees et al., 2014). Here, eDNA‐based detection utilizing common qPCR methods is the main topic of study. It is anticipated that as eDNA workflows advance, more eDNA investigations will employ standardized qPCR techniques to identify target species. This technique makes it feasible to detect species‐specific eDNA from a DNA mixture of different species in the environment and may be used to determine the amount of the target species' DNA through the design of suitable primers and standardization of detection procedures (Shu et al., 2020; Tillotson et al., 2018). In this respect, the species‐specific eDNA analysis is a promising method to discern the nature of eDNA from complex soil habitats.

*Allonychiurus kimi* (Lee, 1973) (Collembola: Onychiuridae) is the dominant collembolan species in the paddy fields of Korea. The influences of temperature, humidity, and nutrition on life table statistics were investigated (Choi et al., 2002), and many ecotoxicology studies were conducted for evaluating ecotoxicity of heavy metals, pesticides, and herbicides (Choi et al., 2008; Son et al., 2007, 2016; Wee, Lee, Kim, Lee, et al., 2021; Wee, Lee, Kim, Son, et al., 2021). In addition, recently, the complete mitochondrial genome of *A. kimi* was reported (Lee, Lee, et al., 2021). *A. kimi* has been employed as a model species for ecotoxicity in the lab rather than in field studies since its population is impacted by different environmental conditions. This species is particularly vulnerable to the harmful impacts of heavy metals in regards to its ability to survive and produce juveniles (Son et al., 2007, 2016). Thus, *A. kimi* has been established as a major bioindicator species in evaluating soil quality and is listed by the International Organization for Standardization (www.iso.org) as an alternative index species (ISO 11267, 1999).

Here, our goal was to use qPCR to establish a species‐specific eDNA technique for *A. kimi* and assess its efficacy utilizing abundances in soil samples with and without *A. kimi*. The results include followings: (1) design real‐time qPCR primers specific to *A. kimi*, (2) test the sensitivity and specificity using eDNA primers from *A. kimi*, and (3) validate species‐specific eDNA analysis on two types of soil samples (soil sample with/without *A. kimi*). Our findings are the first step in establishing sensitive endpoints for eDNA‐based ecotoxicity testing with *A. kimi*.

## 2 MATERIALS AND METHODS

### 2.1 Collembola rearing and maintenance

*Allonychiurus kimi* colonies were first collected from Incheon, South Korea (32.267°N, 127.433°E) on 15 September 1996. Then, this collembolan population has been maintained at the Ecology & Toxicology Laboratory, Korea University, Seoul, South Korea (accession number KUETCOLC001) for approximately 25 years (Lee, Lee et al., 2021). The *A. kimi* colony was maintained in a plastic Petri dish (90 mm × 15 mm (diameter × height) (D × H)) filled with 0.5 cm deep substrate comprise of plaster of Paris, activated charcoal, and distilled water at a ratio of 4:1:4 by volume at 20 ± 1°C with continuous darkness (Wee, Lee, Kim, Son et al., 2021). Distilled water was added periodically, and the plastic Petri dishes were aerated weekly to maintain the fresh condition of the substrate. Brewer's yeast (Sigma‐Aldrich) was added weekly as food. To obtain cohorts of *A. kimi*, eggs laid by hundreds of adults on the same day were transferred to plastic Petri dish with a fresh moist substrate using a fine hair brush. The eggs were hatched after 10 days, and the juveniles were maintained under the same condition for 6 weeks until they develop into adults. Then, the adults were used for all subsequent experiments.

### 2.2 Designing real‐time PCR primers

Based on the previous work from Lee, Lee, et al. (2021), cytochrome oxidase subunit 1 (COI) sequence data of *A. kimi* were obtained from the database of the National Center for Biotechnology Information (NCBI; Accession number: MT975431.1). Obtained 1528‐bp of COI sequences of *A. kimi* were imported into Primer3 software (version 0.4.0) to design species‐specific primers based on following criteria; size of product and primer, melting temperature (*T*_m_) of primer, GC content of designed primer, and avoiding hairpins and dimers (Untergasser et al., 2012; Ye et al., 2012). Seven primer sets acquired from the software were manually evaluated based on the criteria to select primer sets as follows: melting temperature between the primers, avoiding thymine base at the 3′ end of the primer, and cytosine and guanine base at the 3′ end of the primer (Ye et al., 2012). As a result, three species‐specific primer sets for *A. kimi* were selected and subjected to evaluation of specificity in silico and sensitivity and specificity in vitro (Table 1). In this study, the SYBR‐based method was employed in real‐time qPCR analysis because utilizing this method can contribute to making the procedure more affordable and less intensive (Tajadini et al., 2014).

**TABLE 1:** Primer sets designed for target *Allonychiurus kimi*

Primer name	Sequences (5′–3′)	*T*_m_ (°C)	G/C (%)	Amplicon size (nt)	Annealing temperature (Ta) (°C)
AKCO01	334F: GGCCTTGTGGAAAGAGGAGC	60.9	60.00	119	57
	452R: GCCCCAGCTAAGTGAAGGCTA	61.8	57.14		57
AKCO02	349F: GGAGCAGGAACCGGATGAACT	62.1	57.14	106	57
	454R: AAGCCCCAGCTAAGTGAAGGC	62.7	57.14		57
AKCO03	645F: TTTTGACCCAGCAGGAGGGG	62.1	60.00	102	57
	746R: ATCCCAAACCCTGGGAGGAT	60.3	55.00		57

### 2.3 In silico specificity evaluation

To evaluate the efficacy of designated species‐specific primers, first, we conducted in silico PCR analysis against a NCBI database. Using NCBI blast search, a maximum of 5000 whole or partial COI sequences were obtained for each amplicon of three designed primer sets from the standard database optimized for more dissimilar sequences. Then, in silico PCR amplification was performed for each of the three primer sets using the tools provided by the website http://insilico.ehu.es/PCR (Bikandi et al., 2004; Kumar & Chordia, 2015).

### 2.4 Collection of target and nontarget samples for in vitro evaluation

To perform in vitro specificity analysis, seven nontarget species were selected including three hemipteran insect species, which are easily found in the forested areas and four collembola species distributed in South Korea (Bae et al., 2007; Bellinger et al., 1996‐2019; Lee et al., 2013; Lee, Cho, et al., 2019; Lim, 2013; Potapow, 2001). Nontarget hemipteran insects including *Halyomorpha halys* (Stål; Hemiptera: Pentatomidae), *Riptortus pedestris* (Fabricius; Hemiptera: Alydidae), and *Nezara antennata* (Scott; Hemiptera: Pentatomidae) were collected in a soybean field using sweeping net from the Gwangju, South Korea (27.271°N, 127.078°E). The collected specimens were individually placed in 100% ethanol and stored in a deep freezer (IlShinBiobase) until they were subjected to DNA extraction.

For the four nontarget collembola species including *Yuukianura szeptyckii* (Deharveng & Weiner; Collembola: Neanuridae), *Folsomia quadrioculata* (Tullberg; Collembola: Isotomidae), *Folsomia octoculata* (Tullberg; Collembola: Isotomidae), and *Isotomiella minor* (Schäffer; Collembola: Isotomidae) were selected for specificity evaluation. Among the four collembolan species, *Y. sepzeptyckii* were obtained from the colony maintained by Ecology & Toxicology Laboratory, Korea University, Seoul, South Korea (accession number KUETCOLC002; Wee, Lee, Lee, et al., 2021), and the others were collected and identified from the ten soil core samples (5 cm × 10 cm (D × H)) from two forest sites in Chungju, Chungcheong Province (36.979°N, 127.982°E) and Namwon, Jeolla Province (35.429°N, 127.352°E) using Tullgren funnels according to the methods of Lee, Kim, et al. (2021). From the collected soil samples, the specimens were morphologically identified according to the Potapow (2001) and Lee, Cho, et al. (2019) under a stereo‐microscope (SMS 745, Nikon Instruments Inc.). For target species, laboratory‐reared *A. kimi* population was used as described above. Collected specimens were pooled by species up to 20 individuals and stored in 100% ethanol.

### 2.5 In vitro evaluation of species‐specific eDNA primers

Collected target and nontarget specimens were subjected to DNA extraction; DNA samples of whole specimen of hemipteran insects were extracted individually, while DNA samples of collembola species were extracted by pooling 20 individuals in one pool due to the size of the arthropod. Total gDNA was extracted using a AccuPrep® Genomic DNA Extraction Kit (Bioneer) according to the manufacturer's protocol. Each gDNA sample was eluted with 100 μl of nuclease free water. After the DNA extraction, target and nontarget samples were subjected to real‐time quantitative PCR (qPCR) assay using StepOne Plus Real‐Time PCR System (Applied Biosystems). For the specificity evaluation, AccuPower® 2X Greenstar™ qPCR Master Mix (Bioneer) was used according to the manufacturer's protocol under following thermal cycle: 95°C for 10 min followed by 40 cycles of denaturation at 95°C for 10 s, annealing at 57°C for 20 s, and extension at 72°C for 15 s. After the amplification, melting curve analysis was conducted to confirm if each primer set produces single and specific amplicon. The *C*_q_ (quantification cycle) threshold was set at 0.04. According to several studies (Bolotin et al., 2015; Grosdidier et al., 2017; Pfaffl, 2004; Sule & Oluwayelu, 2020), we used the *C*_t_ cutoff at 35 cycles from the qPCR assay to reduce the false‐positive. In general, higher cycle numbers of qPCR increase the probability of nonspecific amplicons. Each of three designed primer sets was subjected to qPCR assay to assess nontarget amplification among the samples. In addition, the COI nt sequences of target and nontarget specimens were obtained from NCBI database. The multiple nt sequences of the COI genes were aligned using Clustal Omega program (https://www.ebi.ac.uk/Tools/msa/clustalo/). Then, the target sites of *A. kimi*‐specific primers were highlighted and confirmed.

For the in vitro sensitivity analysis, each primer set was evaluated against dilution series using DNA extracted from 1, 5, 10, 20, 30, 50, 70, and 100 *A. kimi* individuals. Total gDNAs from each sample were extracted using a AccuPrep® Genomic DNA Extraction Kit according to the manufacturer's protocol. After gDNA extraction, concentration of each sample was measured with a Nanodrop spectrophotometer (Thermo Fisher Scientific). The concentration of gDNA extracted from one individual was 3.1, 5.8 μg/μl from five individuals, 7.9 μg/μl from 10 individuals, 12.9 μg/μl from 20 individuals, 12.7 μg/μl from 30 individuals, 17.3 μg/μl from 50 individuals, 45.7 μg/μl from 70 individuals, and 87.5 μg/μl from 100 individuals. Then, each sample was diluted to 1:10, 1:100, 1:1000, and 1:10,000 and was adjusted to the total volume of 100 μl. After qPCR assay, the *C*_t_ (threshold cycle) and melting curves from the samples of each dilution factors were analyzed.

### 2.6 Collembola rearing experiment for obtaining soil samples

Depth of 10 cm soil was collected from Korea University Farm located in Gyeonggi Province, South Korea (37.346°N, 127.148°E). Collected soil samples were air‐dried at room temperature, subsequently passed through a mesh sieve (diameter 2 mm). Several physicochemical properties were determined using the following: soil particle size by quantifying sand, silt, and clay contents using the pipette method (Gee & Bauder, 1986); soil pH using glass electrode at a soil to water ratio of 1:5 (w/w); and organic matter using the loss on ignition method (Wright et al., 2008). The soil was classified as sandy loam consisting of 54.7% sand, 35.4% silt, 9.9% clay, and 3.6% organic matter content with a pH of 6.71 ± 0.01.

**FIGURE 1:**
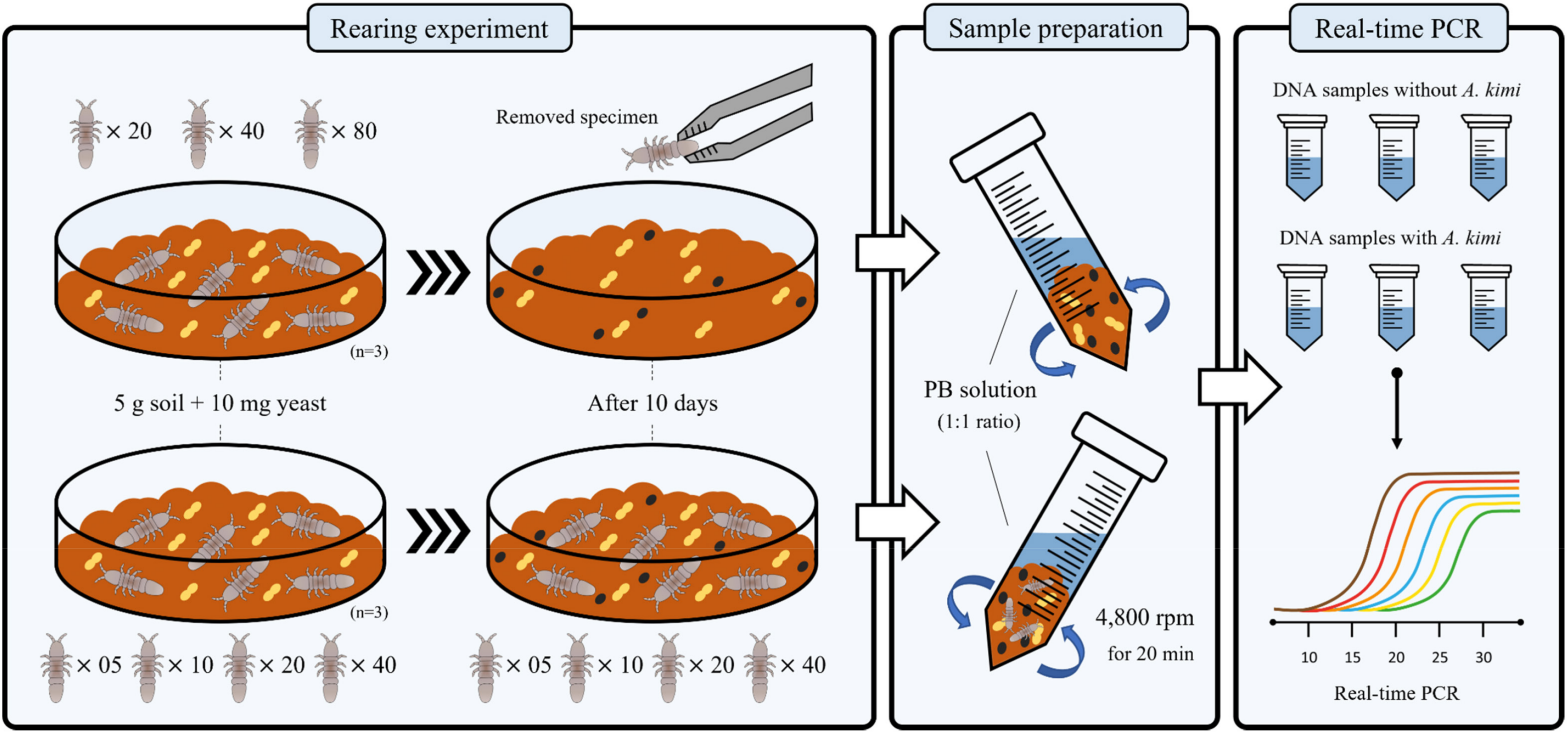
Overall research scheme to evaluate detection efficacy of *Allonychiurus kimi* from eDNA sample using designed primer sets

To obtain the soil sample for qPCR analysis, rearing experiment of collembolan was conducted with the collected soil. Water holding capacity and pH in the soil were adjusted to 60% and 6.5 ± 0.5, respectively (Wee, Lee, Kim, Lee, et al., 2021). The tests were conducted in two sets depending on whether obtained soil samples contain inoculated *A. kimi*. Five grams of soil was placed in a Petri dish (60 mm × 15 mm (D × H)) to depth of 1.5 mm. Approximately 10 mg of dry yeast food was added at the beginning of the test. To collect soil samples with *A. kimi* (intra‐ and extra‐organismal eDNA), 0 (negative control), 5, 10, 20, and 40 individuals were introduced to soil‐containing Petri dish. The test was replicated three times for each sample at 20 ± 0.5°C in continuous darkness. After 10 days, a set of total soil samples were transferred into 15 ml conical tube for the soil samples with *A. kimi* individuals. In contrast, for the only extraorganismal eDNA samples, 0 (negative control), 20, 40, 80 individuals were first introduced to soil‐containing Petri dish. After 10 days later, adult individuals were removed prior to transfer the soil samples into 15 ml conical tubes. Collected soil samples were stored in a −80°C freezer (IlShinBiobase) until eDNA was extracted. The detailed schematic diagram of the rearing experiment is shown in Figure 1.

### 2.7 Detection and quantification of target eDNA from soil sample

Collected soil samples with and without *A. kimi* specimen were subjected to extracellular DNA extraction using modified protocol proposed by Taberlet et al. (2012). First, the saturated phosphate buffer (Na_2_HPO_4_; 0.12 M; pH 8) was added to the soil sample which had the same weight and thoroughly mixed for 5 s to make soil into sludge. Then, sludge was homogenized 5 cycles of 30 s at 5600 *g* with 30 s intervals using Precellys Evolution Homogenizer (Bertin Technologies; Hestetun et al., 2021). Two millilitre of the sludge then collected and centrifuged at 10,000 *g* for 10 min (Hanil Science Industrial). The 600 μl of supernatant was subjected to extracellular DNA extraction using DNeasy® PowerSoil® Pro Kit (Cat. No. 47016; Qiagen), skipping the lysis step and following the manufacturer's protocol. DNA samples were subjected to qPCR assay using designed primer sets with same conditions as described above.

### 2.8 Statistical analysis

Prior to conduct statistical analysis, *C*_t_ values obtained by qPCR from the soil samples with *A. kimi* (*C*_t(with *A. kimi*)_) and without the species (*C*_t(without *A. kimi*)_), were tested for normality using Shapiro–Wilk test (Shapiro & Wilk, 1965). All data set showed normal distribution (*W* > 0.05). A one‐way ANOVA was conducted to determine if the number of inoculated *A. kimi* affected the *C*_t(with *A. kimi*)_ and *C*_t(without *A. kimi*)_ values. After significance was assessed by ANOVA, Tukey's HSD post hoc comparisons were conducted using the LSMEAN option in SAS. In addition, the relationships between *C*_t_‐value and dilution factor and between the *C*_t_‐values and the number of inoculated *A. kimi* (*x*) applied common logarithm were fitted by linear regression: *C*_t_ = *A* + *B*log_10_(*x*). All probability levels used for the statistical significance were *p* < .05. All statistical analyses were conducted using SAS software, version 9.3 (SAS Institute, 2011).

## 3 RESULTS

### 3.1 In silico specificity of species‐specific primers

Designed primer sets of *A. kimi* targets the COI gene showed no nontarget PCR amplicon for all the three primer sets from in silico specificity analysis (Table 1). For AKCO03 primer set, one additional species was matched as nontarget species among the 5000 queries evaluated: *Paulhutchinsonia* sp. (Accession number: MN583785.1). Matched organism *Paulhutchinsonia* sp. reported to be observed only at museum collections in Australia, hence proceeded to in vitro specificity and sensitivity evaluation (Jin et al., 2020).

### 3.2 In vitro evaluation of species‐specific primers

**FIGURE 2:**
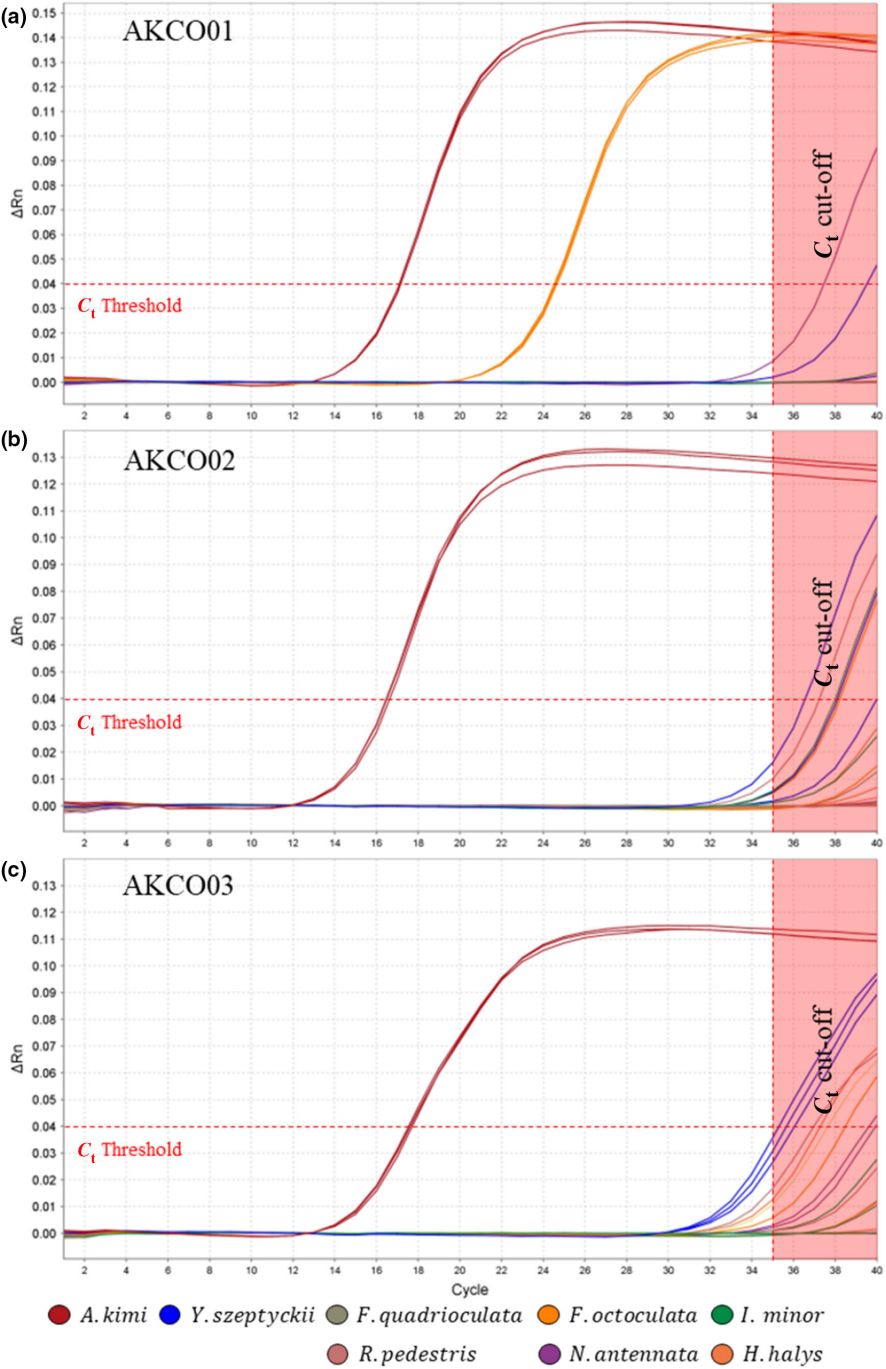
The in vitro specificity analysis of the three designed primer sets (a) AKCO01, (b) AKCO02 and (c) AKCO03, respectively. The *C*_t_ (threshold cycle) threshold was set at 0.04. Red square box indicates cycle cutoff which was used at 35 cycle to determine the positive or negative of tested samples.

**FIGURE 3:**
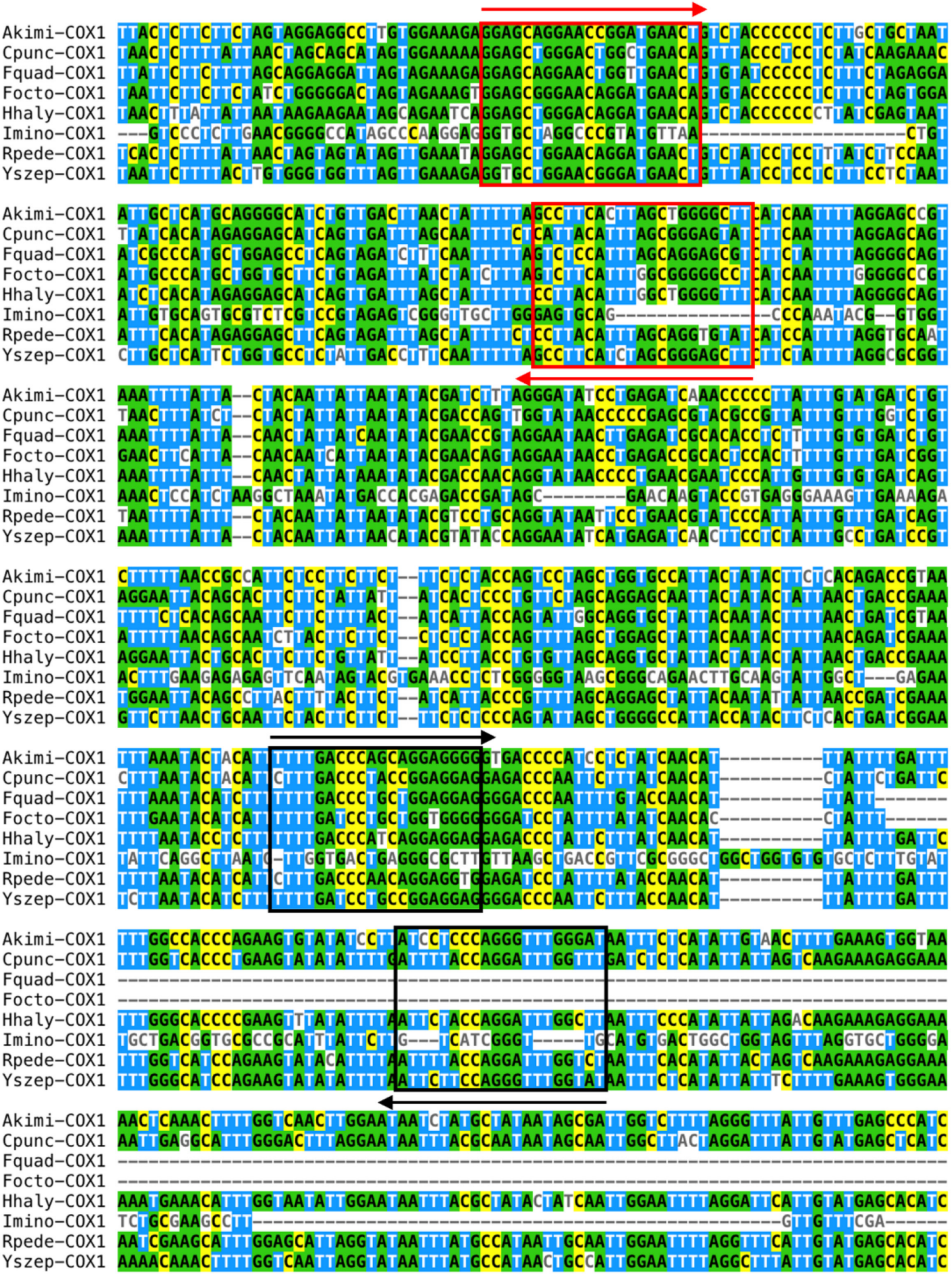
Alignment of the partial nucleotide sequences of cytochrome c oxidase subunit 1 from Akimi‐COX1, *Allonychiurus Kimi* (accession no.: NC_053646.1), Cpunc‐COX1, *Cletus punctiger* (accession no.: NC_050997.1), Fquad‐COX1, *Folsomia quadrioculata* (accession no.: NC_050997.1), Focto‐COX1, *Folsomia octoculata* (accession no.: LC213067.1), Imino‐COX1, *Isotomiella minor* (accession no.: KY230843.1), Rpede‐COX1, *Riptortus pedestris* (accession no.: NC_012462.1), Hhaly‐ COX1, *Halyomorpha halys* (accession no.: NC_013272.1), and Yszep‐COX1, *Yuukianura szeptyckii* (accession no.: NC_054366.1). Quantitative‐PCR target sites are located either in 349–455 n.t. or 645–747 n.t. of Akimi‐COX1 sequences. The red boxes and the black boxes shows the significant sequence polymorphisms in the qPCR targets. The red and black arrows indicate the forward and reverse primer sites for qPCR AKCO02 (red) and AKCO03 (black), respectively.

From the in vitro specificity analysis, first, all designed primer sets showed strong positive for *A. kimi* samples in which *C*_t_ value was recorded below 17 from real‐time PCR assay (Figure 2; Appendix S1). When nontarget amplification was evaluated, no amplification was observed from nontarget hemipteran insect species for all the three designed primer sets. By contrast, among the four nontarget collembola species tested, nontarget amplification was detected from *F. octoculata* for primer AKCO01 (Figure 2a; Appendix S2a), while no amplification was observed for primer AKCO02 and AKCO03 (Figure 2b,c; Appendix S2b,c); all three samples were tested positive for *F. octoculata*, yielding 24.58 ± 0.03 of *C*_t_ value. Hence, primer AKCO01 was excluded for the in vitro sensitivity analysis. By comparing gene alignment data between the target *A. kimi* and nontarget sample using primers AKCO02 and AKCO03, the potential of species‐specific eDNA primer was once more verified (Figure 3).

**FIGURE 4:**
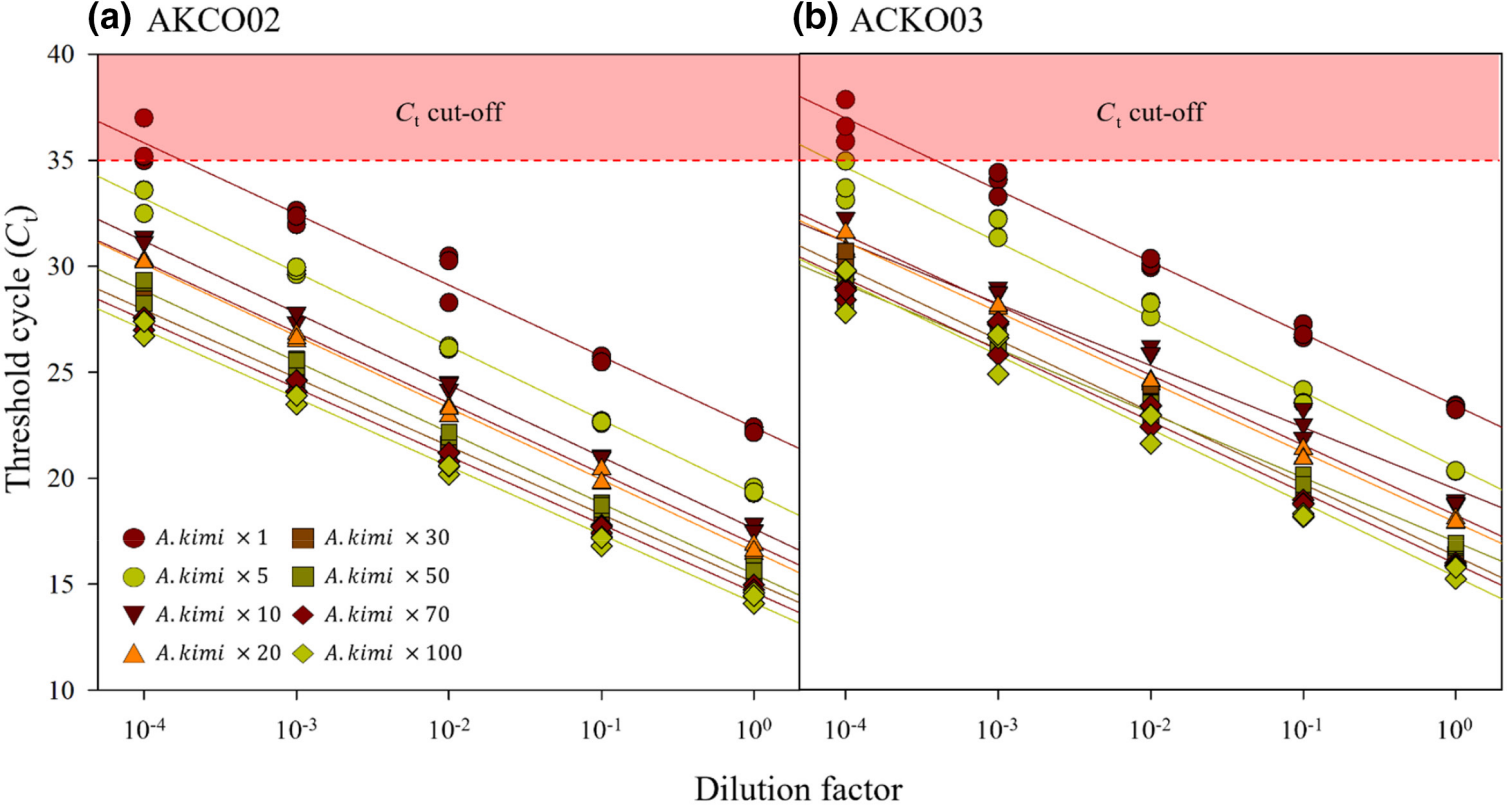
The in vitro sensitivity analysis of the two designed primer sets (a) AKCO02 and (b) AKCO03. Threshold cycle of samples for each group was fitted linear line based on the dilution factors. Red square box indicates cycle cutoff which was used at 35 cycle to determine the positive or negative of tested samples.

The in vitro sensitivity analysis from the two designed primer sets showed that all *A. kimi* samples were positive and formed specimen‐dependent *C*_t_‐values (Figure 4). Also, *C*_t_‐value of pooled samples showed dose‐dependent responses in relation to dilution factors from 1:10^−1^ to 1:10^−4^ ratio. From both AKCO02 and AKCO03 primer sets, negative detection (*C*_t_‐value of >35) was observed from the samples with one *A. kimi* specimen with 1:10^4^ dilution ratio (Figure 4). Nevertheless, significant correlation between *C*_t_‐value and dilution factor was observed from both primer sets when *C*_t_‐values from each dilution factors were fitted linear regression by the number of *A. kimi*; *R* ^2^ values ranged 0.93 < *R* ^2^ < 0.99 and 0.95 < *R* ^2^ < 0.99 for AKCO02 and AKCO03, respectively.

### 3.3 Relationships between the number of inoculated *A. kimi* and the species‐specific eDNA from soil samples

**FIGURE 5:**
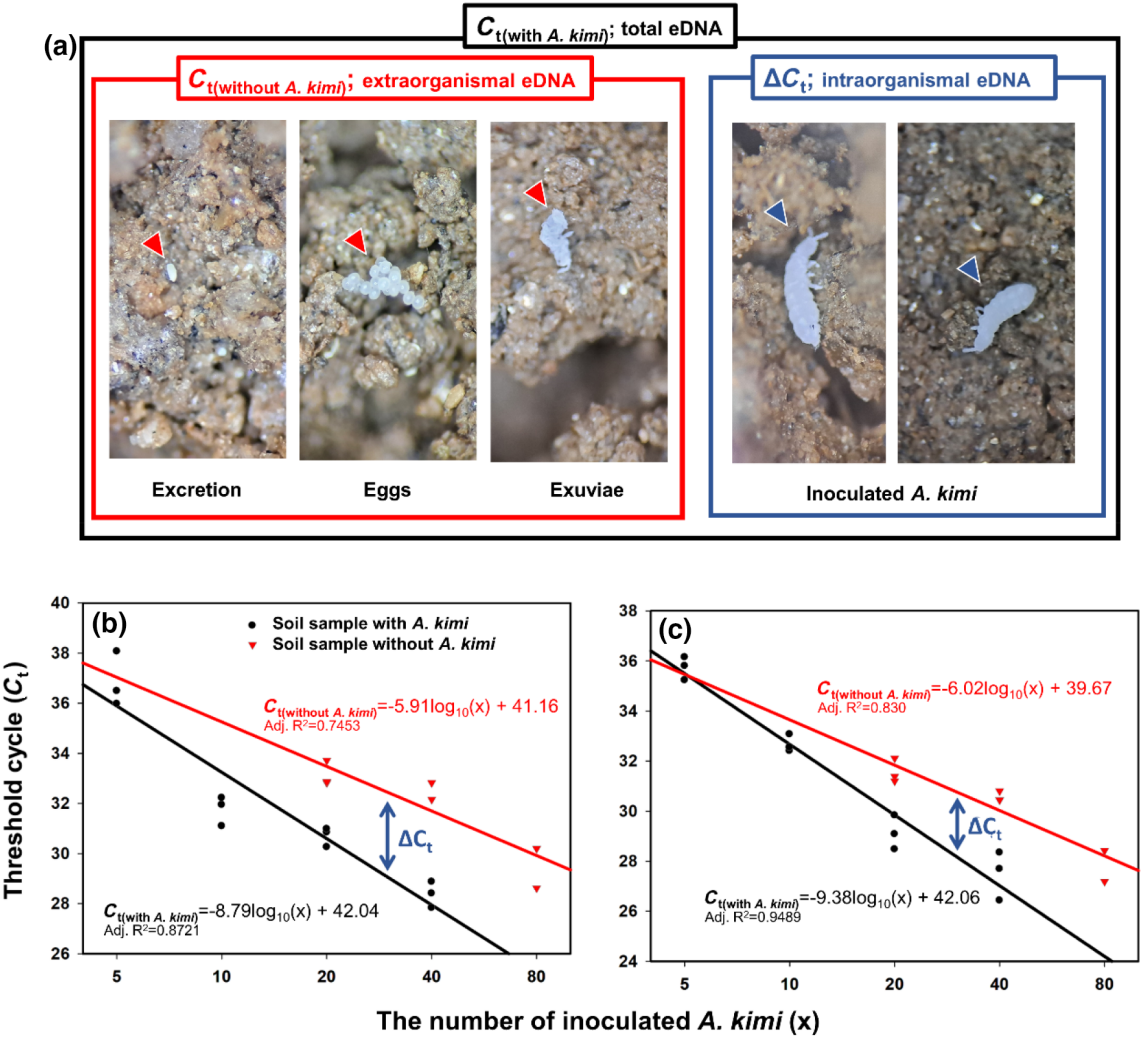
The pictures of the major sources of species‐specific eDNA from which each *C*_t_ value (*C*_t(with *A. kimi*)_, *C*_t(without *A. kimi*)_, and Δ*C*_t_) is derived in the experimental design of this study (a). The threshold cycle (*C*_t_) and linear regressions of the qPCR results of AKCO02 (b) and AKCO03 (c) using soil sample with *Allonychiurus kimi* (*C*_t(with *A. kimi*)_; black circles and black lines), and without *A. kimi* (*C*_t(without *A. kimi*)_; red triangles and red lines). Through the gap between the two estimated regression equations, Δ*C*_t_ of AKCO02 (c) and AKCO03 (d) could be calculated (blue double arrow). In the side of each regression, lines are linear regression equation and adj. *R* ^2^ values. Each *C*_t_ values and the detailed statistics of the regressions are shown in Appendices S3–S5.

The target eDNA concentrations in the soil samples were determined by threshold cycle (*C*_t_) value according to the number of inoculated *A. kimi* (Appendices S3 and S4). The *C*_t (with *A. kimi*)_ and *C*_t (without *A. kimi*)_ were investigated using qPCR from the soil samples either with *A. kimi* or without the species. *C*_t (with *A.kimi*)_ is the value that reflects all species‐specific eDNA of *A. kimi* in the soil including individuals (intra‐ and extra‐organismal eDNA), but *C*_t (without *A.kimi*)_ is the value that only reflects the trace of activity of *A. kimi* in the soil such as molting, excretion, and egg production (extraorganismal eDNA). The difference between *C*_t (with *A.kimi*)_ and *C*_t (without *A.kimi*)_, Δ *C*_t_ was caused by the intraorganismal DNAs of *A. kimi* (Figure 5a). In addition, all the *C*_t_‐values of treatments showed significant differences according to the number of inoculated *A. kimi* individuals (Appendices S3 and S4). In qPCR analysis from the soil sample with *A. kimi* (intra‐ and extraorganismal eDNA), the *C*_t_‐values of AKCO02 primer set ranged from 36.84 ± 1.09 for soil with five *A. kimi*, 31.76 ± 0.59 for soil with 10 *A. kimi*, 30.70 ± 0.38 for soil with 20 *A. kimi*, and 28.37 ± 0.52 for soil with 40 *A. kimi*. For AKCO03 primer set, *C*_t_‐value ranged from 35.73 ± 0.46 for soil with five *A. kimi*, 32.68 ± 0.35 for soil with 10 *A. kimi*, 29.13 ± 0.68 for soil with 20 *A. kimi*, and 27.49 ± 0.97 for soil with 40 *A. kimi*. By contrast, in the soil sample without *A. kimi* (extraorganismal eDNA only), the *C*_t_‐values of AKCO02 primer set ranged from 33.13 ± 0.23 for soil with 20 *A. kimi*, 32.38 ± 0.18 for soil with 40 *A. kimi*, and 29.41 ± 1.12 for soil with 80 *A. kimi*. For AKCO03 primer set ranged from 31.56 ± 0.22 for soil with 20 *A. kimi*, 30.56 ± 0.09 for soil with 40 *A. kimi*, and 27.81 ± 0.87 for soil with 80 *A. kimi*.

Based on these relationships, the *C*_t_‐values were well described by the linear model (Figure 5b,c; Appendix S5). The following linear regressions were performed on *C*_t_‐values obtained by qPCR and logarithm of number of inoculated *A. kimi* individuals; AKCO02: *C*_t (with *A.kimi*)_ = −8.79log10(*x*) + 42.04; *C*_t (without *A.kimi*)_ = −5.91log10(*x*) + 41.16 (Figure 5b), AKCO03: *C*_t (with *A.kimi*)_ = −9.38log10(*x*) + 42.06; *C*_t (without *A.kimi*)_ = −6.02log10(*x*) + 39.67 (Figure 5c). The *R* ^2^ of these regressions ranged from 0.7435 to 0.9489, and AKCO03 showed higher *R* ^2^ than those of AKCO02, which means that *C*_t_‐values of AKCO03 can be better explained than those of AKCO02. The Δ*C*_t_ value can be estimated by these regressions and tends to increase as the number of inoculated *A. kimi* increases.

## 4 DISCUSSION

eDNA‐based biomonitoring has been considered a useful tool for identifying species and estimating biomass in studies where collecting organisms is difficult and impractical (Barnes et al., 2016). As a result, these techniques are frequently employed to assess the condition of the ecosystem and environment. Despite the fact that eDNA has been driving rapid advances in ecology and providing new insights, several issues remain a major challenge to overcome (Bohmann et al., 2014). Since each target organism for research has different ecological characteristics, it is necessary to identify the origin, transport, and degradation of eDNA according to target taxa (Barnes et al., 2016). Our study is a first step towards the study of the origin of the eDNA of soil invertebrates, which are considered robust bioindicator species but have little basic research of eDNA.

In this study, two novel PCR primer sets were designed that can amplify the species‐specific DNA of the soil index species, *A. kimi*. From the sensitivity analysis, we confirmed availability of primer sets which displayed species‐ and dose‐dependent *C*_t_‐values. Low threshold of both primer sets was identified as diluted for 10^−3^:1 ratio for one *A. kimi* included sample. Moreover, conjugated with the previous methods from Taberlet et al. (2012) and Hestetun et al. (2021), we observed clear differences in eDNA quantities extracted from the soil samples with target‐species compared with the soil samples without target species. This result indicates that the identification of intra‐/extraorganismal eDNA in this study should be considered in the ecological context of eDNA of collembolan in soil.

Our study suggests the need to overcome the limitations of existing ecotoxicological risk assessments by developing a novel method to analyze eDNA from soil samples. The results showed that the eDNA from microarthropods in soil does not simply represent the abundance or biomass of collembola individuals in the soil. The total eDNA of *A. kimi* in soil was the sum of intra‐ and extraorganismal eDNA. Among them, extraorganismal eDNA mainly originated from their excrements, eggs, and exuviae which relate to survival, growth, and reproduction. This implies that the amount of eDNA in the soil contains information beyond abundance or density of target species. The amount of eDNA of soil invertebrates has a potential to be more sensitive and integrated index than classical indices such as the number of individuals of indicator species. For a long time, many studies have focused on population of the soil invertebrates as a basic indicator of soil ecotoxicology and ecological risk assessment (Hopkin, 1997). The abundance has been considered a robust index for representing the soil health, but many studies have presented limitations of the existing methods for assessing soil quality through the number of adult and juveniles of Collembola (Lee et al., 2016, 2018; Lee, Son, et al., 2019; Son et al., 2022). First, it is a costly and laborious procedure to count and confirm the results. Moreover, some field experiments require taxonomic knowledge to identify the target taxa (Lee, Kim, et al., 2021). Second, it can be challenging to identify risks that have minor short‐term effects but major long‐term adverse outcomes (Lee et al., 2018). Lastly, there is limitation of existing methods in which although environmental changes and disturbance cause a decrease in the number of molting, hatching rate, and oviposition, it cannot be adequately quantified by classic approaches (Lee, Son, et al., 2019; Son et al., 2022). To overcome these limits, some studies suggested alternative approaches (Fountain & Hopkin, 2001; Kristensen et al., 2004; Lee, Son, et al., 2019). For example, the compressed soil test allows continuous monitoring of molting, number of eggs and hatchability without pretreatment during exposure periods because on the compressed soil collembolans cannot burrow in the soil (Lee, Son et al., 2019). Moreover, some studies suggested more sensitive biomarkers using regulating patterns of protein, composition of fatty acid, and gene expression (Lee et al., 2018; Nota et al., 2008; Wee, Lee, Kim, Son, et al., 2021). Although these alternative approaches and biomarkers overcome the limitations of the existing methods, it is still a laborious procedure to obtain the results. In addition, since sensitive biomarkers are highly related to specific biological traits of individuals, it is difficult to evaluate an integrated risk of population or community. For instance, *Yuukianura szeptyckii*, a biomarker colembolan species employed in ecotoxicology, was used in toxicity experiments. In this experiment, exposure to tebufenozide, one of the insect growth regulators, drastically decreased the expression of numerous proteins associated to glycolysis and energy production (Lee et al., 2018). Thus, those proteins were suggested as a promising index. However, these sensitive biomarkers reflect only the protein level of individuals, not the state of the population in which the individuals are included. Our research approach can easily obtain results from DNA quantitation techniques alone and can provide more sensitive and integrated index than the abundance of the Collembola population.

In our study, the significant finding of intra‐ and extraorganismal eDNA of Collembola in soil suggests that the origin of eDNA should be considered more than other taxa when studying the eDNA of soil invertebrates such as collembolan species. Since the origin of eDNA differs according to the ecological characteristics of the biome, it is necessary to clearly define the origin of eDNA (Barnes & Turner, 2016) in terms of conservation and ecological studies. For example, the studies of eDNA of macroorganisms, the origin of eDNA is mainly defined as traces of organisms such as hair, feces, and other shed biological materials rather than whole organisms (Beja‐Pereira et al., 2009). On the other hand, in cases of microorganisms, the origin of eDNA is considered as the microorganisms themselves, not their trace (Kavehei et al., 2022). In soil‐dwelling collembola, the eDNA quantity, like microorganisms, has been considered as a value that represent the population size (Liu et al., 2022). The findings from our study, however, suggest that eDNA of collembolan have a high variation caused by intra‐ and extra‐organismal eDNA. This is because the eDNA of soil microinvertebrates from the field soil samples will be obtained in a mixed state from various sources and both intra and extraorganismal eDNA which can significantly affect the results based on eDNA analysis. In the eDNA metabarcoding results of the collembola community, DNA of a species not found in actual microscopic examinations is amplified (Saitoh et al., 2016), and some of this may be explained by the extraorganismal eDNA in our study.

Differences in experimental procedures, including sampling, identification, and enumeration of samples from soil samples, can lead to different results for eDNA data analysis. To overcome these challenges, standardized eDNA assays can enable more accurate analysis and also save labor and time for experiments. For example, although the detection and quantification of eDNA makes it easy to determine the distribution and abundance of target organisms in water and soil, changes in eDNA over time are dependent on temperature, pH, and U.V. (Barnes et al., 2014; Troth et al., 2021) and other abiotic factors can affect the quality and quantity of eDNA samples (Pawlowski et al., 2021). Nevertheless, it is still not well known how abiotic factors affect the quality and quantity of eDNA in soil, not to mention biological factors. In addition, the effect of biological and abiotic conditions on eDNA depends on the origin of the eDNA. Therefore, in order to properly interpret the data obtained from eDNA analysis, future studies on the movement and degradation of eDNA according to biological and abiotic factors and the origin of the eDNA are needed.

## AUTHOR CONTRIBUTIONS

**Yun‐Sik Lee:** Conceptualization (lead); data curation (lead); investigation (lead); methodology (lead); writing – original draft (lead); writing – review and editing (lead). **Minhyung Jung:** Conceptualization (equal); data curation (lead); investigation (lead); methodology (lead); writing – original draft (lead); writing – review and editing (equal). **June Wee:** Investigation (equal); methodology (equal). **Yongeun Kim:** Conceptualization (supporting); data curation (supporting); methodology (supporting). **Doo‐Hyung Lee:** Writing – original draft (supporting); writing – review and editing (supporting). **Dong‐Sung Lee:** Methodology (supporting); writing – original draft (supporting); writing – review and editing (supporting). **Taewoo Kim:** Investigation (supporting). **Kijong Cho:** Conceptualization (equal); methodology (equal); supervision (equal); writing – original draft (equal); writing – review and editing (equal). **Cheolho Sim:** Conceptualization (equal); data curation (equal); methodology (equal); supervision (equal); writing – original draft (lead); writing – review and editing (equal).

## Supporting information


Appendix S1‐S5

## Data Availability

Related data are found in Appendix.
