# Supplementary material for: Species‐specific environmental DNA analysis of the index species in soil ecosystem, Allonychiurus kimi (Collembola: Onychiuridae)
Source: Ecol Evol. 2022 Dec 12;12(12):e9598. doi: 10.1002/ece3.9598 (PMC9745010; doi:10.1002/ece3.9598)
Supplement: Supplementary file 1 — Appendix S1‐S5 [file ECE3-12-e9598-s001.docx]

**Appendix**

Appendix 1. Specificity test for designated primer. Gray shading indicates negative detection based on the C_t_ cut-off at 35 cycle.

| Species name | Individual per sample | C*t* value | | |
| --- | --- | --- | --- | --- |
|  |  | AKCO01 | AKCO02 | AKCO03 |
| *Allonychiurus kimi* | 10 (n=3) | 17.09±0.02 | 16.53±0.04 | 17.63±0.04 |
| *Yuukianura szeptyckii* | 10 (n=3) | 39.84±0.12 | 38.22±0.81 | 36.70±0.18 |
| *Folsomia quadrioculata* | 10 (n=3) | Undetermined | Undetermined | Undetermined |
| *Folsomia* *octoculata* | 10 (n=3) | 24.58±0.03 | 39.41±0.48 | 39.50±0.40 |
| *Isotomiella minor* | 10 (n=3) | Undetermined | 39.34±0.53 | Undetermined |
| *Riptortus pedestris* | 1 (n=3) | Undetermined | 39.06±0.76 | 38.99±0.81 |
| *Nezara antennata* | 1 (n=3) | 39.14±0.69 | Undetermined | 39.85±0.09 |
| *Halyomorpha halys* | 1 (n=3) | Undetermined | Undetermined | 39.09±0.73 |


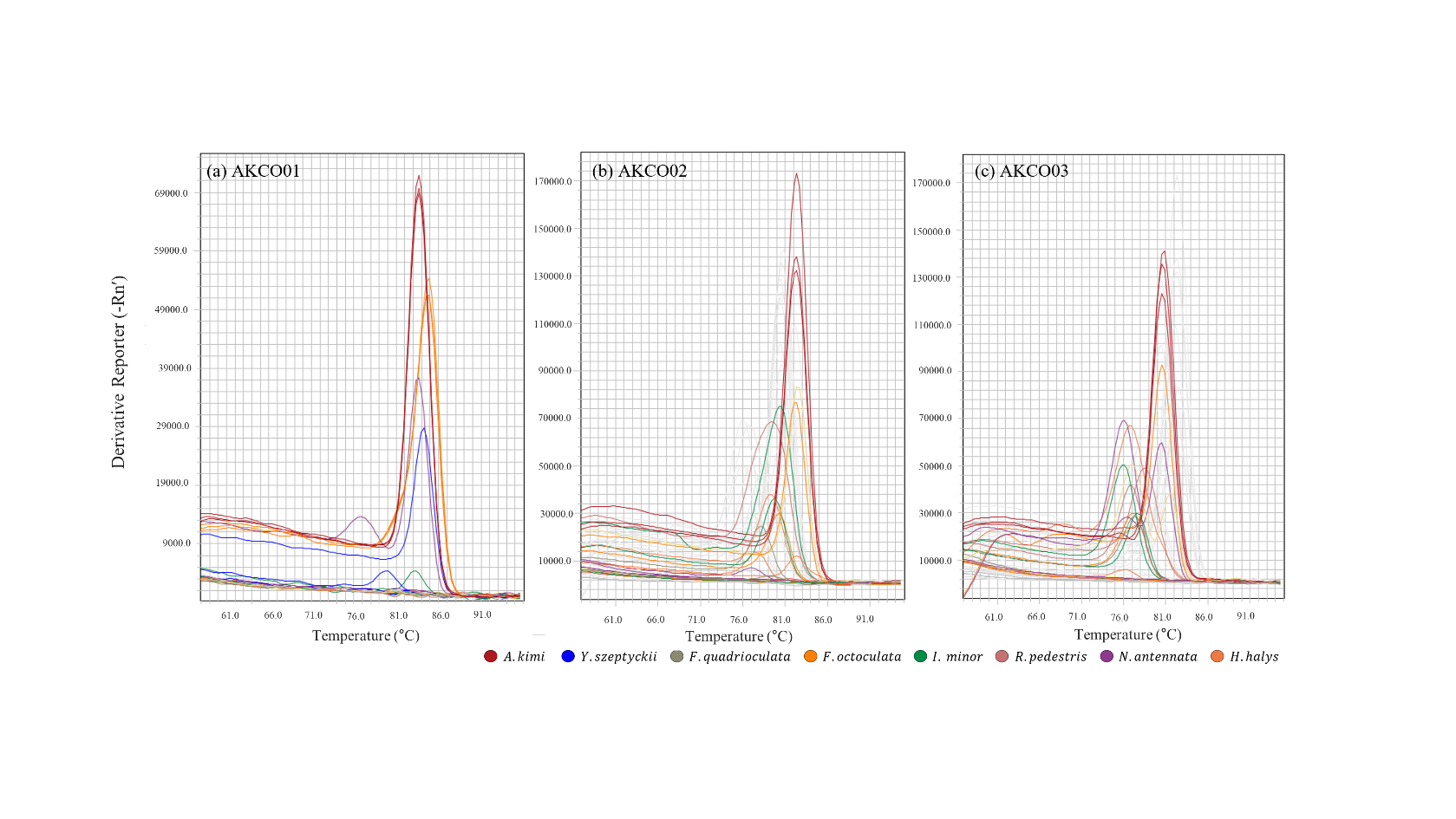


Appendix 2. Melt curve difference of target and non-target observed from the real-time quantitative PCR using designed primer sets (a) AKCO01, (b) AKCO02 and (c) AKCO03.

Appendix 3. Threshold cycle using soil sample without *A. kimi* (Ct_(without_ *_A. kimi_*_)_) and the statistics of ANOVA for each primer. Specimen was removed after the rearing period (10 days). Means within the same column followed by same letters are not significantly different at *p* > 0.05 (Tukey’s HSD test).

| Treatment  (n=3) | C*t* value | |
| --- | --- | --- |
|  | AKCO02 | AKCO03 |
| 5mg soil | Undetermined | 36.94±0.54 |
| 5mg soil + yeast | Undetermined | 38.65±0.38 |
| 5mg soil with 20 *A. kimi* | 33.13±0.23a | 31.56±0.22a |
| 5mg soil with 40 *A. kimi* | 32.38±0.18a | 30.56±0.09a |
| 5mg soil with 80 *A. kimi* | 29.41±1.12b | 27.81±0.87b |
| df | 2, 8 | 2, 8 |
| F | 21.58 | 33.18 |
| *p* value | 0.0035 | 0.0013 |

Appendix 4. Threshold cycle using soil sample with *A. kimi* (Ct_(with_ *_A. kimi_*_)_) and the statistics of ANOVA for each primer. After rearing period (10 days), total DNA was extracted including *A. kimi* specimen. Means within the same column followed by same letters are not significantly different at *p* > 0.05 (Tukey’s HSD test).

| Treatment  (n=3) | C*t* value | |
| --- | --- | --- |
|  | AKCO02 | AKCO03 |
| 5mg soil | Undetermined | 39.80±0.19 |
| 5mg soil + yeast | Undetermined | Undetermined |
| 5mg soil with five *A. kimi* | 36.84±1.09a | 35.73±0.46a |
| 5mg soil with 10 *A. kimi* | 31.76±0.59b | 32.68±0.35b |
| 5mg soil with 20 *A. kimi* | 30.70±0.38b | 29.13±0.68c |
| 5mg soil with 40 *A. kimi* | 28.37±0.52c | 27.49±0.97d |
| df | 3, 11 | 3, 11 |
| F | 78.35 | 93.59 |
| *p* value | < 0.0001 | <0.0001 |

Appendix 5. The statistics of linear regressions for quantification of *A. kimi* based on qPCR results of Primer 1 and 2.

| Statistics | AKCO02 | | AKCO03 | |
| --- | --- | --- | --- | --- |
|  | with *A. kimi* | without *A. kimi* | with *A. kimi* | without *A. kimi* |
| Intercept | 42.04136 | 41.1614 | 42.05507 | 39.668 |
| Standard E | 1.20984 | 2.01588 | 0.78553 | 1.606 |
| T | 34.75 | 20.42 | 53.54 | 24.710 |
| P | <0.0001 | <0.0001 | <0.0001 | <0.0001 |
| Slope | -8.79746 | -590647 | -9.38434 | -6.021 |
| Standard E | 1.00927 | 1.27428 | 0.6553 | 1.015 |
| T | -8.72 | -4.64 | -14.32 | -5.930 |
| P | <0.001 | 0.0036 | <0.0001 | 0.001 |
| R^2^ | 0.8837 | 0.7817 | 0.9535 | 0.854 |
| Adj R^2^ | 0.8721 | 0.7453 | 0.9489 | 0.830 |
